# Relationships of Anemia, Serum Iron, and Serum Copper in Hospitalized Alpacas (*Vicugna pacos*)

**DOI:** 10.1007/s12011-025-04918-1

**Published:** 2025-11-27

**Authors:** Max Kornblum, Johannes Buchallik-Schregel, Petra Röhrig, Antje Polifka, Martin Ganter, Matthias Gerhard Wagener

**Affiliations:** Clinic for Swine and Small Ruminants, Forensic Medicine and Ambulatory Service, University of Veterinary Medicine Hannover, Foundation, Hannover, Germany

**Keywords:** South American camelids, Alpaca, Anemia, Copper, Iron, Trace element

## Abstract

A large number of South American camelids (SAC) presented to a veterinary clinic are diagnosed with anemia. While various causes are known, such as infections with *Haemonchus contortus*, chronic inflammation, neoplasia, gastric ulcers, or deficiencies, little is known about the role of the trace elements copper and iron in SAC. In this retrospective study, the laboratory diagnostic data, in particular the red and white blood count, as well as the findings of the initial clinical examination of 181 alpacas presented at the University of Veterinary Medicine Hannover, Hannover, Germany in the period from January 2020 to September 2023 were therefore analyzed. In addition, iron, copper, transferrin, and ceruloplasmin were determined in the serum collected during the initial examination. The statistical analysis revealed significant correlations between the serum iron content and various degrees of anemia, and also between serum copper content and various degrees of anemia. These observations confirm the need to characterize the iron metabolism of SAC more precisely to improve differentiation between different causes of anemia.

## Introduction

Several South American camelids (SAC) presented to the veterinary practice are diagnosed with anemia [1]. Possible factors discussed causing anemia are endoparasitosis due to *Haemonchus contortus*, *Fasciola hepatica*, chronic inflammation, neoplasia, mineral deficiencies like cobalt-, copper-, and iron deficiency, emaciation, or gastric ulcers [2–5]. However, the characterization of anemia in SAC is difficult due to the special hematologic characteristics of SAC and the lack of criteria for classifying anemia [6, 7]. To our knowledge, there is little information on the relationship between iron (Fe) deficiency anemia and transferrin (Tf), ceruloplasmin (Cp), and copper (Cu). For other species it was already described that Cp, which fulfills an essential storage and transport function for Cu, also plays an important role in iron metabolism [8]. Thus, a low Cu supply can lead to Fe deficiency anemia despite a normal intake of Fe, as it cannot be incorporated into hemoglobin [9] and is therefore not available for hematopoiesis.

Iron as a trace element is part of hemoglobin, which in its biologically active form in the erythrocytes is crucial for the transport of oxygen in the blood [10]. The availability of Fe for erythropoiesis therefore is of great importance. Orally ingested Fe is absorbed mainly by the enterocytes of the duodenum. After oxidation from Fe^2+^ to Fe^3+^ at the basolateral surface by hephaestin, an enzyme containing Cu, and ferroportin-mediated export, it can be bound to transferrin [11]. The expression level of ferroportin is regulated by hepcidin, an acute phase protein whose synthesis is regulated by interleukin-6 and the serum Fe level. It also causes so-called “iron trapping” in macrophages through internalisation of ferroportin [12], thereby reducing the availability of Fe. Cells that have formed transferrin receptors bind the transferrin, internalize it through an endosome and can thus absorb the Fe it contains [11]. The intracellular Fe store is the glycoprotein ferritin, whose synthesis is regulated through a cascade of reactions depending on the intracellular Fe concentration [11]. Since the serum ferritin content correlates with the ferritin present in the cells, it is used for evaluation of iron storage [13].

Copper, on the other hand, is a component of several enzymes that are crucial for Fe metabolism. As previously mentioned, it is part of Cp and also of hephaestin, both multicopper ferroxidases whose function are to catalyze the oxidation process from Fe^2+^ to Fe^3+^ [11]. As the enzymatic function of the oxidases is lacking in the case of Cu deficiency, the availability of Fe decreases. This explains why Cu deficiency has been described as a cause of microcytic anemia in other mammals like cattle [14, 15] just as Fe deficiency. To our knowledge, this phenomenon has not yet been described in SAC. However, due to the mechanisms described, which are strongly conserved in mammals, it appears reasonable to investigate both elements together.

The focus of this study is to find correlations between the trace elements Fe and Cu, markers indicating their regulation, and the severity of anemia. Therefor, the parameters Fe, transferrin, caeruloplasmin, and Cu were determined in the serum of alpaca (*Vicugna pacos*) presented to the clinic and compared with hematologic findings of the animals.

## Materials and Methods

The patient data used for this retrospective study originate from 181 alpacas presented to the Clinic for Swine and Small Ruminants, Forensic Medicine and Ambulatory Service of the University of Veterinary Medicine Hannover, Foundation, Hannover, Germany between January 2020 and September 2023. This includes both sick and healthy animals of both sexes at different ages. The patient data and laboratory results were retrieved from the patient management program “easyVET” (VetZ. EasyVET. Available online at: https://www.vetz.vet/de-de/easyVET, accessed on August 29, 2025) and the laboratory management program “LabControl” (LabControl^®^, Ticono GmbH, Hannover, Germany). The owners gave written informed consent for the scientific evaluation of the data collected.

### Inclusion and Exclusion Criteria

This study involves animals which were presented in the period from January 22, 2020, to August 31, 2023. Reasons that led to exclusion were – an incomplete initial clinical examination, no blood samples were taken on the day of arrival, no red and white blood count was taken, or less than the required serum volume of 700 µL was stored. In the case of animals that were presented more than one time to the clinic within the observed period, only the first recording was considered. Animals that revealed hemolytic serum samples with a hemoglobin concentration > 1.0 g/L in the serum were not included in the evaluation.

### Sample Collection

As part of the initial examination, blood was taken from the jugular vein of the animals using EDTA-, heparin- and serum sample tubes (EDTA Monovette 9 mL K3E; Li-Heparin Monovette 9 mL LH; Serum Monovette 9 mL CAT, Sarstedt AG & Co. KG, Nümbrecht, Germany). A complete red and white blood count was made from the EDTA blood sample using the manual standard methods of the clinic’s laboratory as described by Wagener et al. in 2018 [16].

These included the measurement of the packed cell volume (PCV) by centrifuging EDTA-blood in a microhematocrit tube for 10 min at 10,000×g and reading of the percentage of the erythrocyte column using a template [16]. The erythrocyte count was determined by an analyzer (Celltag α VET MEK-6550, Nihon Kohden Europe GmbH, Kleinmachnow, Germany). The leukocyte count was determined in a Neubauer counting chamber after adding 900 µL of 3% acetic acid to 100 µL of whole blood and incubating for 5 min. The leukocytes were differentiated microscopically in a blood smear stained according to Pappenheim [16].

For the determination of hemoglobin, 2.5 mL cyan solution was mixed with 10 µL whole blood. After 5 min incubation in the dark, the photometric determination took place [16].

In some cases, a reticulocyte count was performed using supravital staining with brilliant cresyl blue. Whole blood was mixed 1:1 with a saturated 5% alkaline brilliant cresyl blue solution. The mixture was incubated for 15 min and a smear was prepared. Percentage of reticulocytes out of a total 1000 erythrocytes were counted [16]. 

The serum tube was centrifuged for 15 min at 2000×g and the supernatant was transferred to a new tube and stored at −20 °C after carrying out the measurements of individual parameters required in the individual cases. For this study, the preserved serum samples were thawed over night at + 7 °C and measurements of the following parameters were carried out:

#### Copper

The Cu concentration in the serum was determined using flame atomic absorption spectrometry (AA Spectrometer Solaar M6 MK II, series number 650904 v1.30 Thermo Electron Ltd., Cambridge, UK in combination with Solaar data station software v11.03). Calibration was performed using copper working standards of 0.1, 0.2, and 0.5 mg/L. These were produced using a copper standard solution (Certipur^®^ Copper standard solution 1000 mg/L Cu Lot.: HC31103786, Merck KGaA, Darmstadt, Germany) through dilution with distilled water. Additionally, 140 mmol/L sodium chloride was added for stabilization (Suprapur^®^ Sodium chloride 99,99 Lot.: B1477206 737, Merck KGaA). In addition, a commercial control serum (ClinChek-Control Ref.: 8880–8882 Lot.: 2062, RECIPE Chemicals + Instruments GmbH, Munich, Germany) was measured. To pass the daily quality check, the determined copper concentration of the control serum had to be within the control range specified by the manufacturer. Calibration was carried out twice daily and one of the working standards was also measured randomized after every 10 serum samples. The accuracy of the measurement method is confirmed and certified by annual participation in a round-robin test (INSTAND e.V., Düsseldorf, Germany). For measurement, the serum was diluted 1:10 with distilled water. Three extinction measurements were carried out as part of each measurement. The result was given in the conventional unit mg/L and converted to the SI unit µmol/L using the conversion factor 15.74 in the following formula modified according to Moritz [17]:

$$\:Copper[mg/L]\times\:15.74=Copper[\mu\:mol/L]$$

#### Iron

For measuring the iron concentration, the test kit FerroZine^®^ LT-SI 0100, Labor + Technik Eberhard Lehmann GmbH, Berlin, Germany was used, which employs the method of dissolving the bound iron from the transferrin, reducing the Fe^3+^ ions formed to Fe^2+^ ions, and binding the Fe^2+^ ions in a complex referred to as ferrozine [18], which was measured photometrically in an analyzer (Cobas Mira Plus^®^,Roche Pharma (Schweiz) AG, Basel, Switzerland). Deviating from the manufacturer’s operating instructions due to the small amount of serum available and after prior testing of the reproducibility of the results, the measurements were carried out with only one fifth (100µL instead of 500µL) of the intended amount of sample material and reagents. The accuracy was checked using both a normal and an abnormal control serum (Lot.:1550UN; Lot1212UE, Randox Laboratories Ltd, Crumlin, United Kingdom) at the beginning of each measurement procedure.

#### Iron-Binding Capacity

For the measurement of iron-binding capacity, the test kit “Eisenbindungskapazität” LT-TI 0100, Labor + Technik Eberhard Lehmann GmbH, Berlin, Germany which includes an iron solution and a precipitation reagent containing MgCO_3_ was used in addition to the previously described method for measuring the iron concentration. The use of this test kit in our laboratory was established by Humann-Ziehank & Hennig-Pauka [19]. The principle consists of adding iron ions (Iron solution R1 Fe^3+^ 500 µg/dL) to the serum until free binding sites of transferrin are saturated and then absorbing the surplus of iron ions with the precipitation reagent. Separating of the precipitates was performed by centrifugation (10,000×g, 10 min Heraeus Christ Biofuge B., Heraeus Holding GmbH, Hanau, Germany) The amount of bound iron was then determined from the supernatant as described for the parameter “iron”. The iron concentration measured after this procedure equates to the total iron-binding capacity (TIBC) [20]. The latent iron-binding capacity (LIBC) was calculated by subtracting the serum iron concentration.

#### Transferrin

The transferrin concentration (Tf) was calculated according to the instructions provided by the test kit manufacturer (LT-TI 0100, Labor + Technik Eberhard Lehmann GmbH, Berlin, Germany) by dividing the total iron-binding capacity by the conversion factor 0.2275 according to the following formula:

$$\:Transferrin\:[mg/dL]=TIBC\left[\mu\:mol/L\right]/0.2275$$

#### Hemoglobin

For the photometric measurement of the hemoglobin content, the hemoglobin was converted to hemiglobin using potassium ferricyanide. In addition, a stable cyanhemiglobin complex was formed using cyanide. The reagent used for this was LT-HG 0100 (Labor + Technik Eberhard Lehmann GmbH) of which 25µL was diluted 1:10 with Aqua bidest. according to the worksheet. The absorption of this complex was then measured after 5 min at 550 nm wavelength by the photometric analyzer Cobas Mira Plus^®^ (Roche Pharma (Schweiz) AG).

#### Ceruloplasmin

Ceruloplasmin concentration was measured using N-dimethyl-p-phenylendiamine-di-hydrochloride, whose formation into a purple-colored product is catalyzed by the ceruloplasmin. The method is based on the investigation of Martínez-Subiela et al. [21].

Measurement was performed using the photometric analyzer (Cobas Mira Plus^®^, Roche Pharma (Schweiz) AG, Basel, Switzerland). A total of 10 µL serum was diluted with 200 µL 0.2 mol/L sodium acetate solution made from 1.64 g sodium acetate (Sodium acetate p.a. unaffected by potassium permanganate, Lot.:381381, Merck KGaA), topped up with aqua bidest. to 100 mL and 10 µL 0.001 mol/L EDTA solution made from 0.372 g EDTA (EDTA Disodium salt 2-hydrate for molecular biology Lot.: 0U013255, AppliChem GmbH, Darmstadt, Germany), which was topped up with aqua bidest. to 100 mL. Then 30 µL 0.02 mol/L N, N-dimethyl-p-phenylendiamine (DPD) solution made from 418.2 mg N-dimethyl-p-phenylendiamine-di-hydrochloride (Lot.:128H2626, Sigma Chemical Co., St. Louis, MO, USA) and topped up with aqua bidest. to 100 mL, was added.

The sodium acetate solution solution was set to a pH value of 6.2 using acetic acid (ROTIPURAN^®^ 100%, p.a., Lot.:09358034, Carl Roth GmbH + Co. KG, Karlsruhe, Germany). The measurement was performed photometrically after 6 min incubation at 550 nm wavelength.

## Statistics

Statistical analyses were generated using R Statistical Software (v4.3.1 2023-06-16 ucrt; R Core Team 2023). Descriptive statistics were given as median and standard deviation. The parameters were analyzed using the Shapiro test for normal distribution. Since our parameters were mainly non-normally distributed, Spearman’s rank correlation coefficient was performed, and the correlation analysis was visualized using a heatmap. Significance level (p) and correlation coefficient (𝜌) were given. The influence of the grouping factors sex, age, degree of anemia, leukocyte count, and neutrophil-to-lymphocyte ratio (NLR) on the measured parameters were investigated using the Kruskal-Wallis test. Respective grouping factors are described below. Parameters for which the statistical analysis with the Kruskal-Wallis test revealed significant (*p* < 0.05) differences were tested with Dunn’s test for multiple comparisons using rank sums as a post-hoc test. The increased risk of false-positive results due to multiple testing was taken into account with Bonferroni correction.

### Reference Values Used

#### Anemia

The animals were categorized into five groups depending on the degree of anemia based on the PCV modified to the suggestions by Wittek and Franz in 2021 [22]:

- Fatal: < 0.10 L/L.
- Severe: 0.10–0.14 L/L.
- Moderate: 0.15–0.19 L/L.
- Mild: 0.20–0.24 L/L
- None: >0.25 L/L.

As only one animal with a PCV of 0.11 L/L was classified as severe, it was added to the moderate group and the severe group was therefore not considered in the statistical analysis.

#### Neutrophil-to-Lymphocyte Ratio (NLR)

As there is little information available on NLR in alpacas, a categorization into three levels of inflammatory stress took place. The threshold values are based on the publication by Zahorec [23] originating from human medicine.

- No signs of inflammatory stress: NLR ≤ 3.0.
- Low to moderate inflammatory stress: NLR > 3.0; ≤11.
- High inflammatory stress: NLR > 11.

#### Age

For the classification of age, we chose five groups whose age limits were based on physiological life expectancy.

- Cria: ≤1 year.
- Subadult: >1year; ≤3years.
- Middle:>3 years; ≤6 years.
- Old:>6 years; ≤11 years.
- Very old: >11 years.

#### Leukocytes

For the leukocyte count we chose the reference range from Dawson et al. [24] between 7.1 and 18.6 G/L.

## Results

### Descriptive Statistics

Results of descriptive statistics are shown in Table 1.

**Table 1:** Number, minimum value, maximum value, median, and mean value of the parameters PCV (packed cell volume), age, leukocytes, NLR (neutrophil-to-lymphocyte ratio), Cu (copper), Cp (ceruloplasmin), Fe (iron), Tf (transferrin), TIBC (total iron-binding capacity), and LIBC (latent iron-binding capacity)

Parameter (Unit)	*n*	min	max	median	mean
PCV (L/L)	181	0.05	0.40	0.27	0.27
Age (days)	175	7	6657	1567	1847.68
Leukocytes (G/L)	181	0.90	60.10	10.80	11.87
NLR	167	0.048	95	4.15	6.37
Cu (µmol/L)	181	0.17	1.14	0.55	0.58
CP (mg/L)	181	0.50	2.64	1.23	1.25
Fe (µmol/L)	181	0.70	57.70	14.10	14.72
Tf (mg/dL)	181	76.48	685.71	201.76	214.90
TIBC (µmol/L)	181	17.40	156.00	45.90	48.89
LIBC (µmol/L)	181	4.50	153.60	33.30	34.17

### Correlation Analysis

**Fig. 1:**
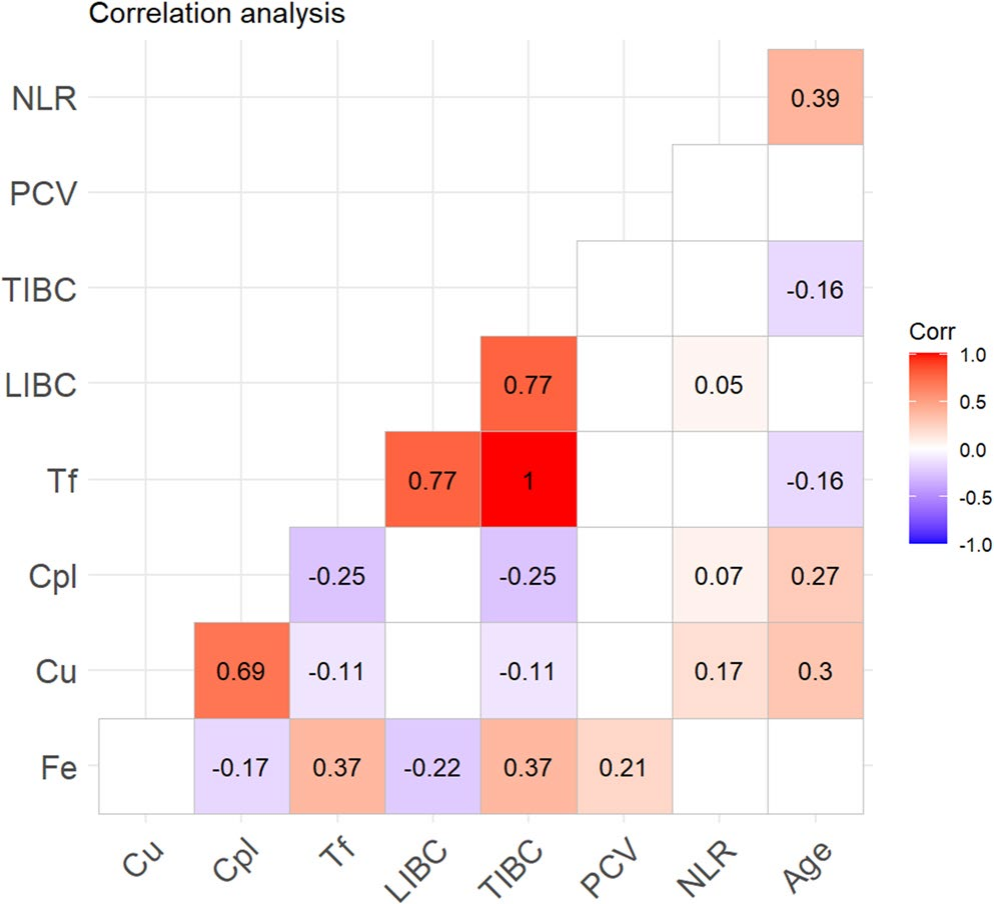
Heatmap with correlational analysis. Correlational results with a significance above the significance level of *p* < 0.05 are blank. The correlation coefficient 𝜌 is characterized by the gradation of color intensity. NLR = neutrophil-to-lymphocyte ratio; PCV = packed cell volume; TIBC = total iron-binding capacity; LIBC = latent iron-binding capacity; Tf = transferrin; Cp = ceruloplasmin; Cu = copper; Fe = iron

The correlation coefficients of the significant correlations are summarized in a heatmap (Fig. 1).

The parameter age correlated significantly with NLR (*p* = 0.008, 𝜌 = 0.39), TIBC (*p* = 0.032, 𝜌 = −0.16), Tf (*p* = 0.032, 𝜌 = −0.16), Cp (*p* = 0.004, 𝜌 = 0.27), and Cu (*p* < 0.001, 𝜌 = 0.30), while there were significant correlations between NLR and LIBC (*p* = 0.037, 𝜌 = 0.05), Cp (*p* = 0.007, 𝜌 = 0.07), and Cu (*p* = 0.006, 𝜌 = 0.17). Furthermore, a significant correlation was detected between Fe and PCV (*p* = 0.004, 𝜌 = 0.21). Numerous significant correlations were found for TIBC, as this parameter correlated with LIBC (*p* < 0.001, 𝜌 = 0.77), Cp (*p* = 0.003, 𝜌 = −0.25), Cu (*p* = 0.024, 𝜌 = −0.11), and Fe (*p* = 0.006, 𝜌 = 0.37). There were also significant correlations between LIBC and Fe (*p* < 0.001, 𝜌 = −0.22). Additionally, Cp correlated with Cu (*p* < 0.001 𝜌 = 0.69) and Fe (*p* = 0.030 𝜌 = −0.17). Correlations with Tf are not listed, as this is a value calculated from TIBC using a factor and the correlations are therefore identical.

### Median (SD) and Significance Depending on Different Categorical Variables

The anemia score had a significant impact on serum Cu, Cp, and Fe (*p* = 0.049). Respective post-hoc analysis revealed that median Cu concentrations were higher in animals showing mild compared to animals expressing fatal anemia (*p* = 0.022). Animals with mild anemia also had higher Cp levels than animals suffering from fatal anemia (*p* = 0.028). Animals diagnosed with mild or moderate anemia had lower Fe concentration in serum compared to animals with physiologic PCV (*p* = 0.038) (Table 2).

**Table 2:** Median and standard deviation [median (SD (standard deviation))] of Cu (copper), Cp (ceruloplasmin), Fe (iron), TIBC (total iron-binding capacity), LIBC (latent iron-binding capacity), and Tf (transferrin) depending on the categorical variable “anemia”. Significant different values in pairwise comparison are marked with distinguishing letters. ^1^Kruskal-Wallis test

Anemia *n* = 181	Fatal *n* = 6	Moderate *n* = 10	Mild *n* = 37	None *n* = 128	*p*-value^1^
Cu (µmol/L)	5.82(3.31)^A^	7.48(2.66)^AB^	9.92(2.97)^B^	8.66(2.71)^AB^	0.027
Cp (mg/L)	109.9(70.14)^A^	185.05(46.93)^AB^	214.9(63.31)^B^	192.5(63.72)^AB^	0.049
Fe (µmol/L)	6.75(10.79)^AB^	7.45(7.14)^A^	11.8(7.1)^A^	15.8(8.15)^B^	0.002
TIBC (µmol/L)	39.9(9.81)	43.65(19.67)	44.1(24.12)	47.7(11.63)	0.240
LIBC (µmol/L)	32.85(7.63)	33.4(20.55)	31.3(24.32)	33.85(12.14)	0.997
Tf (mg/dL)	175.38(43.14)	191.87(86.47)	193.85(106.02)	209.67(51.13)	0.240

There was no effect of sex on measured parameters.

Age had a significant impact on serum Cu concentrations and Cp levels (*p* = 0.019). Lower Cu concentrations were measured in crias than in mature, old, and very old animals (*p* = 0.048), while lower Cp values were measured in crias compared to very old alpacas (*p* = 0.030) (Table 3).

**Table 3:** Median and standard deviation [median (SD (standard deviation)] of Cu (copper), Cp (ceruloplasmin), Fe (iron), TIBC ((total iron-binding capacity), LIBC (latent iron-binding capacity), and Tf (transferrin) depending on the categorical variable “Age”. Significant different values in pairwise comparison are marked with distinguishing letters. ^1^Kruskal-Wallis test

Age *n* = 175	Cria *n* = 26	Young *n* = 43	Mature *n* = 46	Old *n* = 41	Very Old *n* = 19	*p*-value^1^
Cu (µmol/L)	7.4(3.21)^A^	7.87(2.18)^AB^	9.52(2.82)^B^	9.44(2.78)^B^	10.07(3.13)^B^	0.006
Cp (mg/L)	151.5(71.36)^A^	187.7(56.28)^AB^	201.7(72.39)^AB^	194.7(58.26)^AB^	236.2(53.85)^B^	0.019
Fe (µmol/L)	15.7(8.66)	14.2(8.21)	12.75(9.2)	14.1(6.93)	12.1(7.78)	0.414
TIBC (µmol/L)	52.65(24.32)	45.9(14.78)	48.15(13.43)	44.1(10.88)	40.5(10.48)	0.086
LIBC (µmol/L)	34.55(28.06)	33.8(15.42)	35(11.12)	30.5(11.82)	28.6(8.25)	0.384
Tf (mg/dL)	231.43(106.89)	201.76(64.95)	211.65(59.04)	193.85(47.84)	178.02(46.07)	0.086

The leukocyte level had a significant impact on serum copper (*p* = 0.027). In animals with leukocytopenia, higher median Cu concentrations were measured compared to animals having physiologic leukocyte counts (*p* = 0.045) (Table 4).

**Table 4:** Median and standard deviation [median (SD (standard deviation))] of Cu (copper), Cp (ceruloplasmin), Fe (iron), TIBC (total iron-binding capacity), LIBC (latent iron-binding capacity), and Tf (transferrin) depending on the categorical variable “Leukocytes”. Significant different values in pairwise comparison are marked with distinguishing letters. ^1^Kruskal-Wallis test

Leukocytes *n* = 181	Leukocytopenia *n* = 23	Within reference range *n* = 137	Leukocytosis *n* = 21	*p*-value^1^
Cu (µmol/L)	10.55(2.81)^A^	8.18(2.74)^B^	8.97(3.27)^AB^	0.027
Cp (mg/L)	208.3(62.52)	189.2(60.73)	222.3(79.56)	0.091
Fe (µmol/L)	10.9(8.27)	14.2(8.04)	13.3(8.81)	0.120
TIBC (µmol/L)	42(12.28)	47.4(15.84)	42(13.58)	0.055
LIBC (µmol/L)	32.1(10.53)	33.8(16.59)	31(14.93)	0.695
Tf (mg/dL)	184.62(53.99)	208.35(69.65)	184.62(59.71)	0.055

## Discussion

The overall difference among groups between the degree of anemia and serum iron was, as expected, significant. Also the fact that the observed differences between certain levels of anemia and serum copper concentration were significant overlaps with the findings of Van Saun et al. [9] who already discussed the connections between copper and anemia. However, the overall differences among groups found between serum copper and leukocytes confirm the need to characterize the trace element metabolism of SAC more precisely to differentiate between anemia associated with primary iron deficiency and such anemia in the context of chronic inflammation in which the low blood iron content is downregulated by increased expression of the acute phase protein hepcidin [25]. Passler et al. already proved serum iron concentration as a marker of inflammation in alpacas [26]. Regarding the significant pairwise differences between certain levels of anemia and serum copper it has to be considered that iron metabolism is closely linked to copper metabolism, since ceruloplasmin, which fulfills an essential storage and transport function for copper, also has an important enzymatic function in iron metabolism [8]. Bivalent stored iron is oxidized to trivalent iron by the catalyst ceruloplasmin [9] to then be bound to transferrin and transported in the blood [8]. Therefore, a lack of catalysis due to low copper supply induces symptoms comparable to iron deficiency [9]. In this context, Hawkey & Gulland [27] also found hypochromic anemia and occasionally neutropenia in llamas with copper deficiency. The erythrocytes of the affected animals showed anisocytosis, poikilocytosis and hypochromia. Another study by Andrews [28] reported of two llamas with plasma copper levels of 1.3µmol/L and 2.5µmol/L, respectively (one of the animals had already been given a copper-containing medication orally several days before the measurement), and poor condition. They were diagnosed with anemia, which showed improvements in red cell count, hemoglobin concentration, and PCV after copper supplementation. Therefore, the significant differences between the level of anemia and serum copper found in our study could lead to the conclusion that a copper measurement should also be carried out as a routine procedure when examining anemic alpacas. However, Pechová et al. [29] regarded plasma copper concentration not as a predictive indicator for copper intake of alpacas as they found contrary results when they divided 299 alpacas from 18 farms into three groups based on the calculated daily intake of copper and zinc. They found that the animals with the lowest daily copper intake (on average 8.1–10.8 mg/day for a 75 kg animal) had the highest median plasma copper with 8.46 µmol/L, while the animals with low amounts of supplementation had a median of 6.99 µmol/L and animals with high supplementation had 7.65 µmol/L. This could be due to the fact that, as Pechová et al. stated, serum/plasma copper concentration does not represent the supply status, but rather the transport pool containing copper bound to amino acids and ceruloplasmin as a form of copper that has been exported from the liver, which is the main organ of copper storage [29]. It therefore seems comprehensible, as shown for other animals, for example, cattle by López-Alonso et al. [30] and sheep by Humann-Ziehank et al. [31], that the serum/plasma copper level and liver copper level do not correlate well with each other. Therefore copper supply can be better obtained by determining it in the liver tissue, which, however, is a very invasive method and more suitable for post-mortem examination [30].

Ceruloplasmin is synthesized as an acute phase protein in the liver [32]. It contributes to a large proportion of blood copper due to its seven copper atoms per molecule [9]. The blood copper content and also the measurement of ceruloplasmin can therefore vary greatly not only as a result of Cu supplementation, but also as a result of infections [9].

When differentiating between anemia caused by inadequate iron absorption and anemia caused by chronic inflammation, it is common in human medicine to measure soluble iron in the blood, iron in ferritin storage, transferrin, and transferrin saturation in addition to the hepcidin level [33]. To the best of our knowledge, hepcidin measurement has not been carried out in SAC so far. This would improve our understanding of the iron metabolism of SAC. However, in human medicine it has been described that hepcidin binds to the iron export channel ferroportin and prevents the release of iron into the blood through its internalization [33, 34]. Ferroportin is found on enterocytes and also on macrophages [33], which makes oral supplementation of iron less effective [33]. Nevertheless, these interactions have yet to be shown for SAC.

Consideration of reticulocytes in relation to erythrocytes can help to characterize anemia as regenerative or non-regenerative [35, 36]. However, interpreting regeneration requires reference values, which vary for SAC and are completely missing for alpacas to date [7]. For llamas Al-Izzi et al. [37] referred 0- 0.8% as a normal reticulocyte count and addressed differences in age and sex. Fowler and Zinkl [38] established a reference interval (0–0,4%) for adult llamas. For all llamas regardless of age and sex the reference interval was 0 to 2.4%. Reynafarje et al. [39] reported a mean value of 1.4% reticulocytes in healthy alpacas kept at high altitudes, the comparatively high percentage was assumed to display a higher turnover.

For the assessment of regeneration, Wagener et al. [7] suggested using values from other species as an orientation. For dogs this would be >1% as indicator for a mild regeneration and >20% reticulocytes for marked regeneration [40].

In the case of the blood smears considered in this study, additional reticulocyte count was only conducted on specifical request. This was done in 17 animals (9.4% of the animals of this study). The reticulocyte counts exceeded 1% in 13/17 animals (76.5%), but only 2/17 (11.8%) had more than 20% reticulocytes. However, it must be taken into account that these 17 animals are not representative for the study population.

The median serum iron level in animals with a reticulocyte count of < 20% was 4.6 umol/L, which is below the reference values given by Dawson et al. [41] (13.43–34.20 µmol/L) and Stanitzig et al. [42] (male adults: 16.23–78.73 µmol/L, female adults: 9.57–63.81 µmol/L).

It therefore seems possible to conclude that a lack of iron available for erythropoiesis is often a cause of the poor regenerative capacity of anemia in alpacas.

In a study by Schaefer & Stokol [43] in dogs indices of reticulocyte cell size and hemoglobin content were found to be valuabe for differentiating between anemia due to iron deficiency and anemia due to inflammation and other causes. However, transferring this to SAC can prove difficult, as some hematologic analyzers do not have a specific profile for SAC and errors can occur during analysis, particularly due to the ellipsoid erythrocyte shape [16, 44]. Nevertheless, in a previous study we found significant positive correlations between serum iron and MCV as well as MCH, and also negative correlations between serum iron and MCHC in a herd of healthy female alpacas kept under controlled conditions in northern Germany [45].

One parameter that may be easier to implement in routine diagnostics would be the NLR. The NLR can be increased due to inflammation and has established itself as a useful parameter in SAC, as it is easier to measure [46]. However, the correlation between NLR and age shows the need to take age dependency into account when evaluating NLR. Since, to our knowledge, there are no reference values for SAC that take age dependency into account, the use of this parameter is limited.

Another parameter of interest regarding the iron balance is stainable iron. While stainable iron in bone marrow has established itself as the gold standard for determining iron reserves [43], its measurement is far more invasive. Transferrin saturation, on the other hand, can be calculated using the values already collected, and provides information about the percentage of transferrin binding sites filled [33] and thus about the iron storage. Dawson et al. established reference values for alpacas from 22 to 60% transferrin saturation in serum [41]. However, the comparability when applying different measurement methods needs to be considered. To avoid errors in the interpretation of the measurement data, the time course must be taken into account as well. In humans, in the case of insufficient iron intake, ferritin decreases first, indicating depleted iron stores, and then transferrin saturation decreases as a sign of the inability to mobilize sufficient amounts of iron [33].

### Limitations

When interpreting the results, it must be considered that the population studied were alpacas presented to the clinic for various reasons and there is no control population kept and fed under controlled conditions. Therefore, the correlations found cannot be generalized to the entire population or to sick animals in general.

Due to the fact that reticulocyte counts were only available for a small number of animals, it was not possible to classify the anemia as regenerative or non-regenerative for statistical analysis. This would have enabled a more systematic evaluation, but cannot be changed due to the retrospective nature of the study.

For measurement of iron and iron-binding capacity, test kits intended for human serum were used, as it can be assumed that the chemical process of iron ionization is species-independent. The conversion factor for human transferrin was used because there was no such factor for alpacas available. However, transferrin is an evolutionarily highly conserved protein whose fundamental structure hardly differs between mammalian species [47, 48]. Therefore, since to our knowledge there is no specific conversion factor for transferrin, the use of the human conversion factor seems a justifiable approach.

## Conclusions

Further prospective studies, possibly including artificially created iron or copper deficiency, would be necessary to apply the existing knowledge about copper and iron metabolism and respective regulatory mechanisms in humans and other animal species to alpacas. However, this would be of great practical relevance, as in contrast to the areas of origin in South America, SAC are mainly kept as hobby animals in Germany and there are different concepts regarding the use of compound and supplementary feeds when feeding the animals [49], which can easily lead to feed-related deficiencies or overfeeding and even intoxication [50].

Nevertheless, the evaluation shows that the copper and iron balance should be considered when interpreting the hematologic parameters. This would enable conclusions to be drawn concerning the pathogenesis and consequently the appropriate course of treatment to be taken.

## Data Availability

The datasets analyzed during the current study are not publicly available due to data protection regulations. Data are available from the corresponding author on reasonable request.

## Abbreviations

SAC: South American camelids
PCV: Packed cell volume
NLR: Neutrophil-to-lymphocyte ratio
Cp: Ceruloplasmin
Cu: Copper
Fe: Iron
Tf: Transferrin
TIBC: Total iron-binding capacity
LIBC: Latent iron-binding capacity
MCH: Mean corpuscular hemoglobin
MCV: Mean corpuscular volume
MCHC: Mean corpuscular hemoglobin concentration
SD: Standard deviation
